# New URJC-1 Material with Remarkable Stability and Acid-Base Catalytic Properties

**DOI:** 10.3390/polym8020044

**Published:** 2016-02-05

**Authors:** Pedro Leo, Fernando Martínez, Guillermo Calleja, David Briones, Lukasz Wojtas, Gisela Orcajo

**Affiliations:** 1 Department of Chemical and Energy Technology, Chemical and Environmental Technology, Mechanical Technology and Analytical Chemistry, ESCET, Rey Juan Carlos University, c/Tulipán s/n, 28933 Móstoles, Spain; pedro.leo@urjc.es (P.L.); fernando.castillejo@urjc.es (F.M.); guillermo.calleja@urjc.es (G.C.); david.briones@urjc.es (D.B.); 2 Department of Chemistry, University of South Florida, 4202 East Fowler Avenue, Tampa, FL 33620, USA; lwojtas@usf.edu

**Keywords:** URJC-1, MOF, catalyst, stability, acid-basic properties

## Abstract

Emerging new metal-organic structures with tunable physicochemical properties is an exciting research field for diverse applications. In this work, a novel metal-organic framework Cu(HIT)(DMF)_0.5_, named URJC-1, with a three-dimensional non-interpenetrated **utp** topological network, has been synthesized. This material exhibits a microporous structure with unsaturated copper centers and imidazole–tetrazole linkages that provide accessible Lewis acid/base sites. These features make URJC-1 an exceptional candidate for catalytic application in acid and base reactions of interest in fine chemistry. The URJC-1 material also displays a noteworthy thermal and chemical stability in different organic solvents of different polarity and boiling water. Its catalytic activity was evaluated in acid-catalyzed Friedel–Crafts acylation of anisole with acetyl chloride and base-catalyzed Knoevenagel condensation of benzaldehyde with malononitrile. In both cases, URJC-1 material showed very good performance, better than other metal organic frameworks and conventional catalysts. In addition, a remarkable structural stability was proven after several consecutive reaction cycles.

## 1. Introduction

Metal–organic framework (MOFs) materials have been intensively exploited in terms of using a considerable variety of organic ligands containing oxygen, nitrogen, sulfur, or phosphorus as donor atoms to coordinate the metal or metal clusters [1]. Particularly, carboxylate-based MOF materials containing the metal–oxygen coordination have exhibited high crystallinity, remarkable specific surface areas, and tunable pore size and shape. Nevertheless, carboxylate-based MOF materials typically possess low chemical stability in dilute acidic and basic media and are sensitive to moisture [2], being easily hydrolyzable and, consequently, not suitable for many industrial applications. In this sense, azole compounds are considered promising organic ligands for the construction of MOFs due to their great diversity [3,4] and high coordination ability for bridging metal ions, providing strong metal–nitrogen interactions [5,6,7,8]. In addition, *N*-substituted azole compounds like *N*-substituted tetrazole heterocycles, can present intrinsically basic properties [9].

Likewise, transition metal ions are mostly used to produce stable MOF structures due to their ability to easily bind the oxygen and/or nitrogen atoms from the organic ligands and their inherent acid catalytic properties [10]. In the last years, copper-based MOF materials with unsaturated metal centers as Lewis acid sites have been studied in several catalytic reactions, like *O*-arylation of phenols with nitroarenes [11] and Friedel–Crafts acylation [12,13]. Knoevenagel condensation between carbonyl (C=O) and activated methylene groups has also been explored as a feasible catalytic application of MOF materials [14]. Knoevenagel reactions can be catalyzed by bases, acids, or both functionalities [15]. Several authors have reported a positive synergetic effect between neighboring acid and basic sites for UiO-66-NH_2_ [16] and IRMOF-3 with uncoordinated Zn defects as acid sites [17]. Thus, the inclusion of dual acid and basic sites in MOF materials are of potential interest for catalytic applications.

In this context, the aim of this work is to synthesize a new microporous copper-based MOF material with enhanced stability and plausible dual acid-basic properties, by using a non-commercial azole-based ligand previously studied by Dinca *et al.* [18] and our group in the synthesis of ITF-1 material [4].

## 2. Experimental Section

### 2.1. Synthesis of 1H-imidazol-4,5-tetrazole Ligand

**Figure 1:**
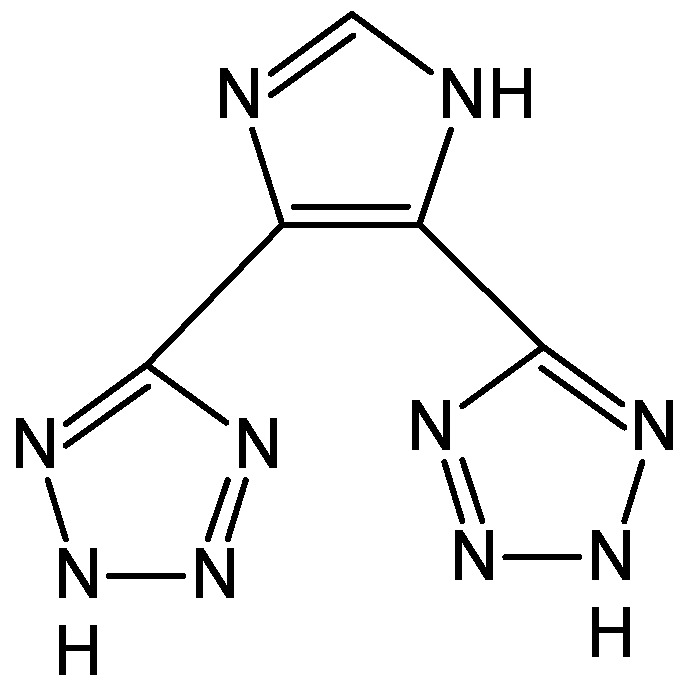
Organic ligand 1H-imidazol-4,5-tetrazole.

1H-imidazol-4,5-tetrazole (HIT, Figure 1) was used as the organic ligand for the preparation of novel URJC-1 material. As HIT is not commercially available, it was prepared following a modified recipe given by Demko *et al.* [19]. Typically, 1H-imizole-4,5-dicarbonitrile (20 mmol), sodium azide (1.43 g, 22 mmol), and zinc bromide (4.50 g, 20 mmol) were added to deionized water (40 mL) in a round-bottom flask. This solution was refluxed under vigorous stirring for 24 h. Thereafter, the resultant white solid was filtered and washed with deionized water several times. Then, the solid was treated in nitric acid aqueous solution (pH = 1) under stirring for 1 h at 85 °C. Finally, the resultant tetrazole-based ligand was filtered, washed with deionized water, and dried at 110 °C for 6 h.

### 2.2. Synthesis of URJC-1 Material

URJC-1 (University Rey Juan Carlos No. 1) material was prepared by mixing 1H-imidazol-4,5-tetrazole (HIT) as the organic ligand and Cu(NO_3_)_2_·xH_2_O as the inorganic source in an acidified solution of *N*,*N*′-dimethylformamide (DMF) and acetonitrile (CH_3_CN) as solvents. The molar synthesis composition was: 1Cu:1HIT:193DMF:287CH_3_CN:109HNO_3_. This mixture was heated at 150 °C for 20 h using a heating rate of 1.5 °C/min. After decanting the mother liquor, the resultant solid product was washed several times with equimolar DMF:CH_3_CN solution and finally activated at 200 °C under high vacuum for 4 h.

### 2.3. Physicochemical Characterization Techniques

Single-crystal X-ray diffraction (XRD) data for URJC-1 were measured on a Bruker D8 Venture PHOTON 100 CMOS system (USF, Tampa, FL, USA) equipped with a CuKα INCOATEC Imus micro-focus source (λ = 1.542 Å). Indexing was performed using *APEX2* [20] (difference vectors method). Data integration and reduction were performed using SaintPlus 6.01 [21]. Absorption correction was performed by the multi-scan method implemented in SADABS [22]. Space groups were determined using XPREP implemented in APEX2 [20]. The structure was solved using SHELXS-97 (direct methods) and refined using SHELXL-2013 [23] (full-matrix least-squares on F^2^) contained in APEX2 [20,23], WinGX v1.70.01 [23,24,25,26] and OLEX2 [23,27]. Non-hydrogen atoms of the framework were refined anisotropically. The disordered, DMF (occupancy ≈ 0.5) has been refined using restraints and with isotropic approximation. The rest of the disordered solvent have been refined as O atoms (possibly water molecules). All hydrogen atoms were placed in geometrically-calculated positions and included in the refinement process using a riding model with isotropic thermal parameters: Uiso(H) = 1.2 Ueq(–CH, –NH), 1.5 Ueq(–CH_3_). Crystal data and refinement conditions are shown in Table S1. Powder X-ray diffraction (PXRD) patterns were obtained in a Philips XPERT PRO (URJC, Mótoles, Spain) using CuKα (λ = 1.542 Å) radiation. In all the cases the samples were grounded in order to avoid the effects of preferred crystal orientation.

Elemental analysis was carried out using an analyzer type ELEMENTAR VARIO EL III, equipped with a thermal conductivity detector. Fourier transform-infrared spectra (FT-IR) were recorded for powder samples in a Varian 3100 Excalibur Series spectrometer (URJC, Mótoles, Spain) with a resolution of 4 cm^−1^. Scanning electron microscopy (SEM) images and micro-elemental analysis (EDS) were obtained on a Philips XL-30 ESEM (URJC, Mótoles, Spain) operated at 200 kV. Simultaneous thermogravimetry and derivative thermogravimetry analyses (TGA/DTGA) using a TA Instruments SDT 2860 apparatus (URJC, Mótoles, Spain) were carried out under a nitrogen atmosphere with an N_2_ flow of 100 mL·min^−1^ at a heating rate of 5 °C·min^−1^ up to 900 °C. Argon adsorption–desorption isotherms were measured at 87 K on an AutoSorb equipment (Quantachrome Instruments, URJC, Mótoles, Spain). Samples were previously evacuated *in situ* under high vacuum (<10^−7^ bar) for 4 h at 200 °C. The micropore surface area values were calculated by using the Brunauer–Emmett–Teller (BET) model. The pore volume was estimated by using the Dubinin–Raduskevich equation. Non-local DFT calculations were used to estimate the pore size distribution using a kernel equilibrium model of slit pore, Ar-carbon at 87 K. Skeletal density was measured at room temperature with a He flow, using Micromeritics Accupyc 1340 apparatus (URJC, Mótoles, Spain).

### 2.4. Catalytic Tests

Friedel–Crafts acylation of anisole with acetyl chloride and Knoevenagel condensation of benzaldehyde with malononitrile were selected as acid and basic catalyzed-type reaction tests. Both reactions were carried out in a round bottom flask placed in a silicone bath under nitrogen atmosphere. For Friedel–Crafts acylation, anisole (10 mmol), acetyl chloride (10 mmol) catalyst (25 mg), and nitrobenzene solvent (10 mL) were charged at room temperature and heated up to 120 °C [12]. In the case of Knoevenagel condensation, malononitrile (5.1 mmol) and catalyst (48 mg) were added to DMF (10 mL) at room temperature, and then heated up to 80 °C. Once the temperature was stabilized, benzaldehyde (5.1 mmol) was added [28]. The stirring was fixed for all runs at 700 rpm in order to avoid diffusional limitations. GC-3900 Varian chromatograph equipped with a CPSIL 8CB capillary column (30 m × 0.25 mm, film thickness 0.25 mm) and a flame ionization detector (FID) was used to monitor reactants conversions and products yields for Friedel–Crafts acylation and Knoevenagel condensation, respectively. *p*-MAP yield was estimated as the product between anisole conversion and the selectivity to *p*-MAP product.

## 3. Results and Discussion

### 3.1. Physicochemical Characterization of URJC-1 Material

**Figure 2:**
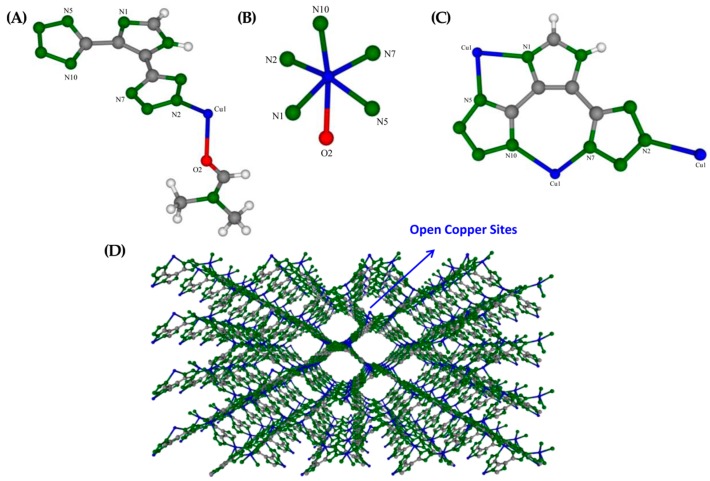
(**A**) Asymmetric unit of URJC-1 structure; (**B**) copper coordination; (**C**) ligand coordination; (**D**) and three-dimensional view of URJC-1 structure free of solvent. Cu: blue, C: grey, N: green, and O: red.

The crystalline structure of URJC-1 material was solved by the single crystal X-ray diffraction technique resulting in a three-dimensional non-interpenetrated **utp** or (10,3)-d topological network [29]. A molar composition of Cu(HIT)(DMF)_0.5_ was determined for the as-synthesized material. The asymmetric unit of the URJC-1 material comprises a copper ion linked to one ligand (HIT) and one DMF solvent molecule (Figure 2A). The copper ion is connected to five nitrogen atoms belonging to tetrazole and imidazole rings of the HIT ligand and one oxygen atom of a carbonyl group of the DMF molecule (Figure 2B). This copper ion exhibits a distorted octahedral CuN_5_O geometry confirmed by the planar Cu–N distances in the range of 1.999(8)–2.031(10) Å for N1, N2, N5, and N7, and axial Cu–N10 and Cu–O distances of 2.171(10) Å and 2.340(2) Å, respectively. The bond angles of the metal environment are in the wide range of 77.8(7)–103.37(4)°. Concerning the HIT ligand coordination (Figure 2C), three copper ions are connected to one HIT molecule. Two coppers are linked to nitrogen atoms of tetrazole rings (N7 and N10) and imidazole and tetrazole rings (N1 and N5, respectively). The third copper atom is connected to nitrogen N2 atom of a tetrazole ring. The crystal data found for the as-synthesized URJC-1 material are: *M*r = 314.25; orthorhombic, Pn21a; *a* = 8.6577(6), *b* = 9.0478(9), *c* = 14.9973(11), *V* = 1174.79 Å^3^; *Z* = 4; *Dc* = 1.77 g/cm^3^. More details of the structural unit of URJC-1 material were included in Figure S1 and Table S1.

The crystallographic data also evidenced a one-dimensional rhombohedral pore system along the [100] direction with dimensions of *ca.* 12.0 × 9.0 Å. The removal of the solvent molecules generated unsaturated copper sites accessible from the porous system, as potential Lewis acid sites. Additionally, non-substituted nitrogen atoms of tetrazole heterocycles also provide Lewis basic sites to the porous structure (see Figure S2). The three-dimensional view of the packed URJC-1 structure along the [100] axis free of solvent is shown in Figure 2D.

**Figure 3:**
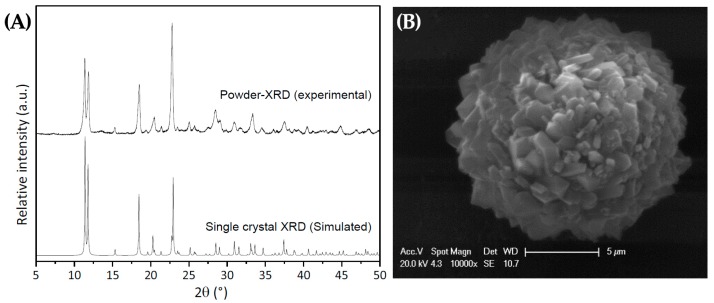
(**A**) XRD patterns of powder URJC-1 sample and simulated from single-crystal data; (**B**) SEM micrographs of the as-synthesized URJC-1 sample.

In order to confirm the purity of the crystalline phase of the URJC-1 material, X-ray diffraction patterns of the bulk powder sample and the simulated pattern from the crystallographic data of the single crystal technique were compared (Figure 3A). As it can be seen, the location and intensity of the main reflections of both patterns match quite well, endorsing that the crystalline structure resolved by single crystal X-ray diffraction is uniquely present in the bulk sample. SEM micrographs of the as-synthesized URJC-1 material shows sphere crystalline particles with uniform size of *ca.* 5–10 μm (Figure 3B).

**Figure 4:**
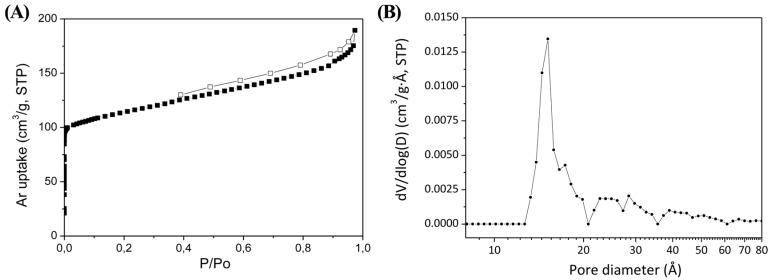
(**A**) Ar adsorption–desorption isotherms at 87 K; and (**B**) pore size distribution estimated by non-local DFT of the activated URJC-1 sample.

The porosity of the copper-based MOF material was measured by argon adsorption/desorption isotherms at 87 K (Figure 4A). The URJC-1 material displayed a characteristic type I adsorption–desorption isotherm of a microporous material with a BET specific surface area of 408 m^2^/g and a pore volume of 0.24 cm^3^/g at *P*/*P*_0_ of 0.998. Additionally, a pore size distribution centered at 14.8 Å was estimated using the NL-DFT method applied for an Ar–carbon kernel at 87 K based on a slit pore model (Figure 4B). The mean pore size was slightly higher than that found from the crystallography data. Crystal density of the activated sample was experimental measured by He pycnometry resulting 1.45 g/cm^3^.

The copper content of the powder URJC-1 material was also determined by ICP-OES. The metal content of the activated sample was 22.6%. This value is very close to the theoretical content (23.8%) calculated from the crystallographic molecule, excluding coordinated DMF solvent molecules. Thus, it is evident that DMF molecules were successfully removed from the crystalline framework, confirming the presence of unsaturated metal centers with potential activity as Lewis acid sites. Therefore, URJC-1 material appears as a very promising porous material with dual acid and basic sites, due to the co-existence of accessible unsaturated copper centers and non-coordinated nitrogen atoms of the HIT ligand, respectively.

### 3.2. Thermal and Chemical Stability of URJC-1 Material

**Figure 5:**
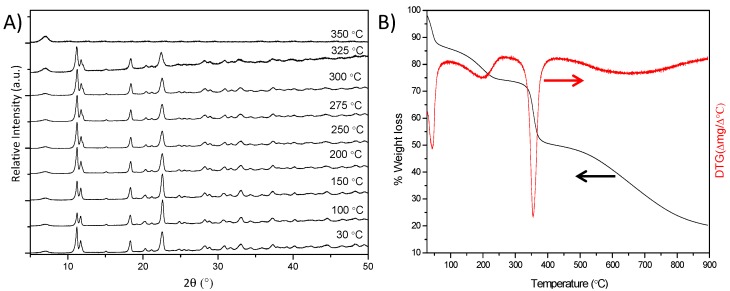
(**A**) *In-situ* temperature-dependence PXRD patterns and (**B**) TG/DTG analyses of URJC-1 material.

Figure 5 shows *in situ* temperature-dependence PXRD patterns and TG/DTG analyses in N_2_ atmosphere of URJC-1 sample. *In situ* PXRD patterns at different temperatures display a remarkable structural integrity of URJC-1 sample up to 325 °C (Figure 5A). Likewise, TG/DTG analyses also confirm this stability with a remarkable weight loss in the range of 340–380 °C and a maximum DTG peak centered at 350 °C (Figure 5B), which is associated with the decomposition of the HIT ligand and subsequent structure collapse. It is also noticeable the presence of two other weight losses (*ca.* 80 and 160 °C), which are attributed to the elimination of the synthesis solvents, acetonitrile/water and *N*,*N*-dimethylformamide, respectively.

**Figure 6:**
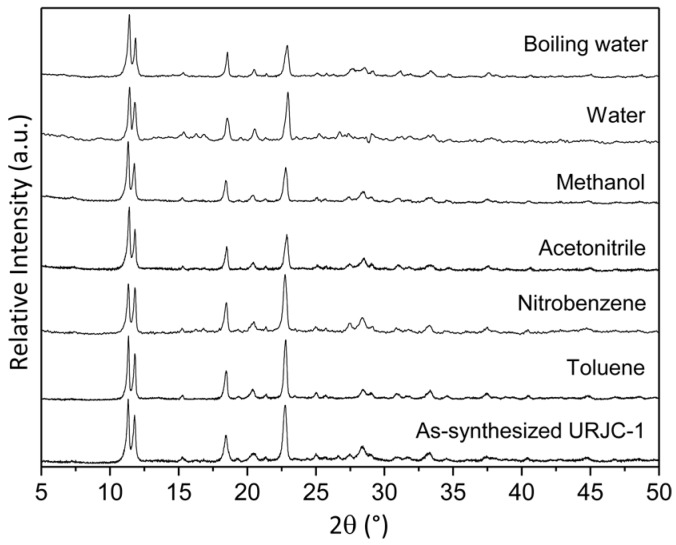
PXRD patterns of the URJC-1 material after suspension in different solvents.

The chemical stability of URJC-1 material was tested by dispersion of the solid sample in different organic solvents. The solvents were selected according to their different polarity (polarity index of toluene is 2.3 and that of water 10.2). Figure 6 shows the PXRD patterns of URJC-1 material after dispersion in each solvent during 24 h at room temperature. As it is observed, the crystalline structure remained intact after the chemical stability tests, evidencing a strong robustness of the copper–imidazole/tetrazole framework. Additionally, the URJC-1 material also maintained its integrity when it was immersed in boiling water for 1 h (also shown in Figure 6).

**Figure 7:**
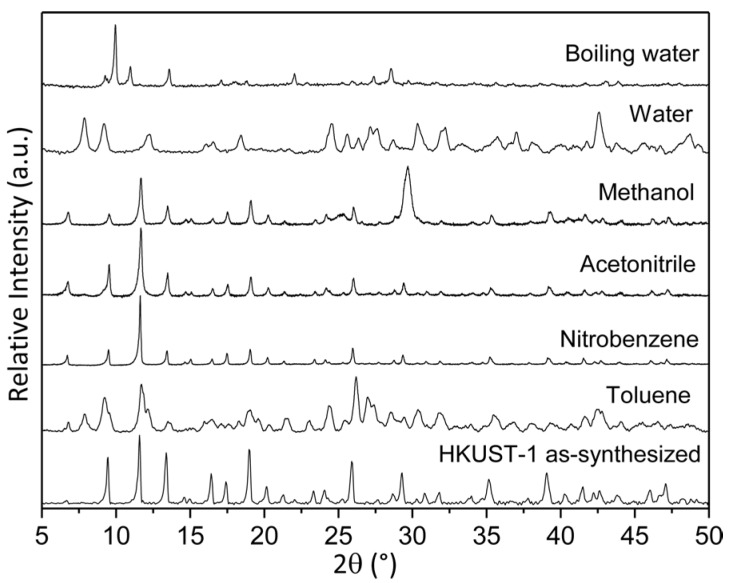
PXRD patterns of HKUST-1 material after suspension in different solvents.

Similar stability tests were also carried out for one of the most extended Cu–carboxylic-based MOF, the commercial HKUST-1 (or Basolite C-300). In this case, the characteristic crystalline phase of HKUST-1 material was drastically modified after 24 h in water, toluene, and methanol, maintaining it only in acetonitrile and nitrobenzene (Figure 7). Additionally, the structure also collapsed in boiling water after 1 h. These results clearly evidenced a minor structural strength of this MOF structure based on 4-coordinate Cu^2+^ with oxygen linkages, as compared to the URJC-1 structure based on 5-coordinate Cu^2+^ and nitrogen linkages.

**Figure 8:**
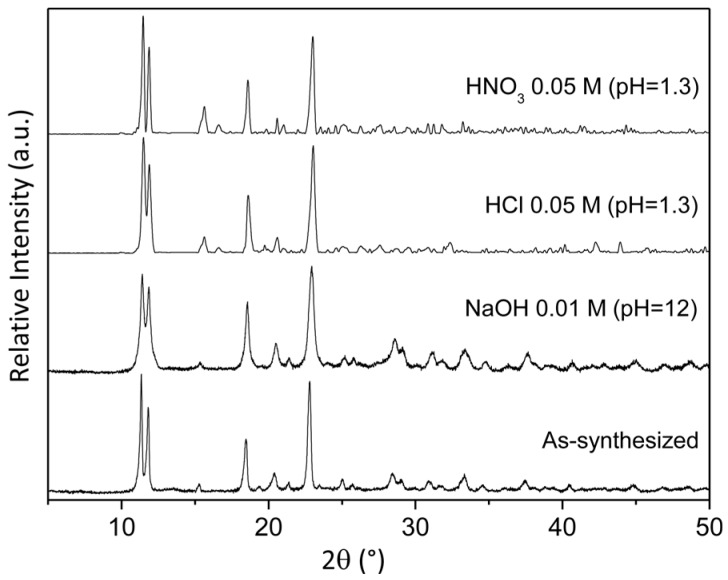
PXRD patterns of URJC-1 material after suspension in acid and basic solutions.

Finally, the stability of the URJC-1 material was also studied under strong acidic and basic conditions using HCl and HNO_3_ solutions (0.05M, pH = 1.3) and NaOH solution (0.01M, pH = 12). Figure 8 shows PXRD patterns of URJC-1 samples after 1 h at room temperature, evidencing that the crystalline URJC-1 framework was also stable under acid and basic agents. From these results, it has been attested that the URJC-1 material provides not only a crystalline microporous framework with promising acid and basic properties but also a remarkable thermal and chemical stability, which make it very attractive for catalytic applications.

### 3.3. Catalytic Activity of URJC-1 Material

The activity of the URJC-1 material was assessed in the reaction of anisole acylation with acetyl chloride and in the Knoevenagel condensation of benzaldehyde with malononitrile, being both typical catalytic tests for acid and basic-catalyzed reactions, respectively.

**Figure 9:**
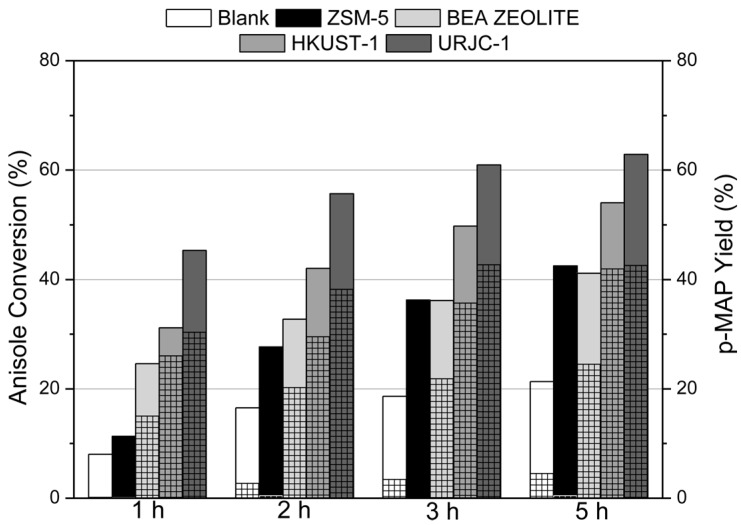
Catalytic performance of URJC-1 material and other catalytic materials in the acylation of anisole. Normal columns indicate anisole conversion; dense fill columns indicate *p*-MAP yield. On the other hand, PRXD of URJC-1 and HKUST-1 materials before and after acylation reaction were performed in order to evaluate the catalyst stability of MOF materials (Figure 10). Interestingly, the URJC-1 material did not undergo any modification of the main diffraction peaks. In contrast, the structure of Cu-carboxylic MOF material (HKUST-1) collapsed after reaction according to the PXRD patterns of the recovered sample. Therefore, the higher stability of the URJC-1 structure enables the reusability of this material for successive catalytic runs.

In the case of the acylation of anisole, URJC-1 material was studied and compared with other catalysts typically used for this reaction, like Cu-based MOF material (HKUST-1) and some conventional microporous zeolitic materials (Beta and ZSM-5), using the same operation conditions of previous works [12,30]. Figure 9 shows the catalytic performance in terms of anisole conversion and *p*-methoxyacetophenone (*p-*MAP) yield as the final product for all materials, including a blank experiment with no catalyst. All the catalysts showed a better performance than the blank experiment. Among the tested materials, URJC-1 exhibited the best catalytic activity. Note that URJC-1 has lower surface area (408 m^2^/g) than HKUST-1 (708 m^2^/g). In these materials, the catalytic activity is related to the active role of unsaturated copper centers as Lewis acid sites. Regarding the zeolitic materials, it was also remarkable not only the higher anisole conversion for Cu-based MOF materials but also the enhancement of *p*-MAP yield. Nevertheless, taking into account the reaction rate referred to the number of catalytic sites, also known as turnover frequency (TOF), MOF materials revealed lower values of TOF as compared to zeolitic samples of this work (see Table S2) [31]. This fact is probably associated to certain diffusion constraints of the organic reactants to get the metal sites as consequence of stronger interactions of reactants within the metal organic framework. Another hypothesis could be also the different nature of the catalytic sites of assessed samples (aluminum sites in the case of ZSM-5 and BETA materials and copper sites in the case of MOFs). Thus, the higher activity of MOF materials, and in particular of URJC-1, is due to the high concentration of active acid sites per gram of catalyst.

**Figure 10:**
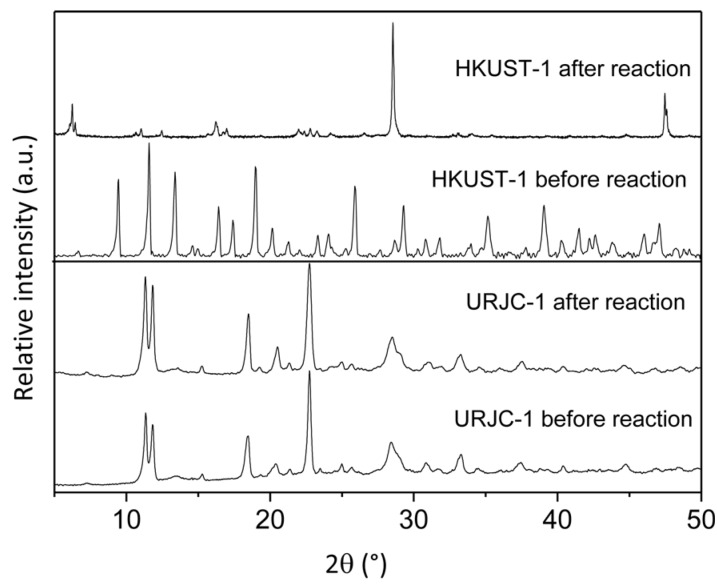
PXRD patterns of URJC-1 and HKUST-1 materials before and after the acid catalyzed acylation reaction.

**Figure 11:**
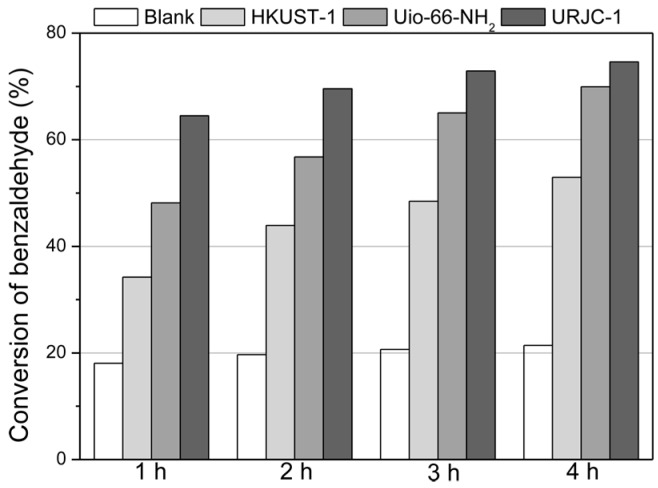
Catalytic performance of the URJC-1 material and other heterogeneous catalysts in Knoevenagel condensation of benzaldehyde and malononitrile reaction.

The URJC-1 material was also tested as a catalyst in the Knoevenagel condensation of benzaldehyde with malononitrile to produce 2-benzylidenemalononitrile (2-BM). In this case, the catalytic performance of the URJC-1 material was compared to HKUST-1 and UiO-66-NH_2_ MOF materials which provide basic sites from oxygen atoms of the metal-carboxylated linkages (HKUST-1) and amino groups of the organic ligand (UiO-66-NH_2_). Figure 11 illustrates the conversion of benzaldehyde for all materials as well as a blank experiment without any catalyst, in the same operation conditions of previous works [28]. It is remarkable that high selectivity toward 2-BM product (above 99%) was obtained in all cases.

The basicity of the oxygen atoms from the metal-carboxylated linkages of HKUST-1 material [32] seems to be too weak to activate the benzaldehyde condensation, leading to conversion of benzaldehyde only slightly greater than the blank experiment. On the other side, UiO-66-NH_2_ material enhanced significantly the catalytic performance. This fact has been attributed to the synergetic effect of neighboring structural zirconium acid sites to amino-containing groups of the ligand [16]. The catalytic performance of the URJC-1 material was even superior to that shown by the UiO-66-NH_2_ sample. Herein, the synergetic effect of nitrogen basic sites of the ligand containing tetrazole groups and unsaturated copper acid sites could be also responsible of its remarkable activity [16].

The turnover frequency parameter was also estimated for the three MOF materials in order to compare the catalytic activity per active site (Table S3). It has to be noted that this is a gross estimation since the different nature of basic sites for each MOF material and also the assumption of total accessibility of all active sites (non-coordinated nitrogen atoms from tetrazole groups for URJC-1, basic oxygen atoms of the metal–carboxylate linkages for HKUST-1, and amino groups for UiO-66-NH_2_. Amino-containing MOF showed the highest TOF value, followed by URJC-1 and, finally, HKUST-1 materials, possibly due to the higher basicity of the amino groups of UiO-66-NH_2_ in comparison with the nitrogens from tetrazole groups of URJC-1 or oxygens from carboxylated ligands of HKUST-1.

**Figure 12:**
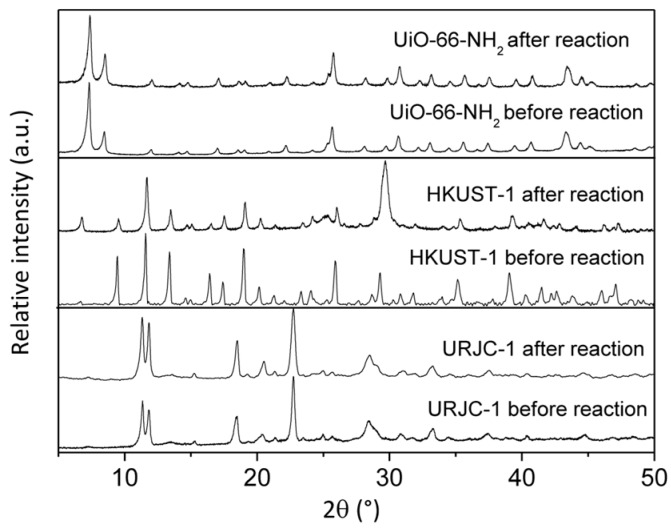
PXRD patterns of URJC-1, UIO-66-NH_2_, and HKUST-1 materials before and after Knoevenagel condensation of benzaldehyde with malononitrile.

Similarly to the other catalytic tests, the stability of the three materials was assessed by comparison of PXRD patterns of the fresh and recovered material after reaction (Figure 12). PXRD patterns of URJC-1 and UiO-66-NH_2_ materials revealed good stability under the conditions of Knoevenagel condensation reaction. In contrast, the HKUST-1 material showed a partial collapse of the structure, as shown by the disappearance of some characteristic diffraction peaks of its structure.

### 3.4. Reusability of URJC-1 Material

**Figure 13:**
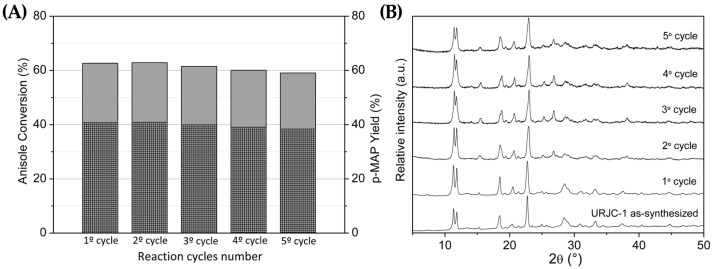
(**A**) Catalytic performance of URJC-1 in several acylation reaction cycles. Normal columns indicate anisole conversion; dense fill columns indicate *p*-MAP yield; and (**B**) X-ray diffraction patterns of fresh and used URJC-1 catalyst after several anisole acylation reaction cycles.

Reusability of URJC-1 as a catalyst was also assesed in both reactions, carrying out until five consecutive reaction cycles at the same operation conditions described in the experimental section. The structural stability of the materials was evaluated after each reaction cycle by PXRD. The results displayed in Figure 13A evidence a catalytic activity practically constant in terms of anisole conversion and *p-*MAP yield for five reaction cycles of 5 h each. Additionally, PXRD patterns of the catalyst after each reaction cycle confirm the stability of URJC-1 structure, demonstrating again a remarkable chemical stability (Figure 13B).

**Figure 14:**
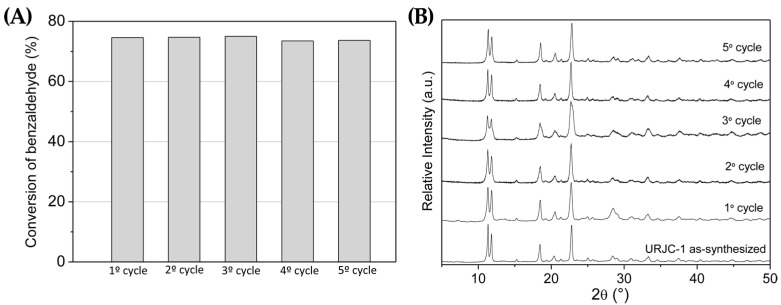
(**A**) Catalytic performance of URJC-1 in several Knoevenagel condensation reaction cycles; and (**B**) X-ray diffraction patterns of fresh and used URJC-1 catalyst after several Knoevenagel condensation reaction cycles.

Regarding the Knoevenagel condensation reaction, the URJC-1 material also maintained its activity, in terms of benzaldehyde conversion, and structural integrity for five consecutive cycles of 4 h each, as it is observed in Figure 14A,B, respectively.

## 4. Conclusions

A novel metal-organic framework, named URJC-1, has been synthesized and characterized by single crystal X-ray diffraction. This material, based on the linkage of 1H-imidazol-4,5-tetrazole (HIT) with copper (II) ions, exhibits a 3D structure with 1-D microporous channels of 12 × 9 Å in the [100] direction and BET specific surface area of 408 m^2^/g and a pore volume of 0.24 cm^3^/g. This material displays dual acid and basic sites as a result of accessible unsaturated copper sites and non-coordinated nitrogen atoms from the tetrazole groups of the ligand. The URJC-1 material has exceptional thermal and chemical stability in different organic solvents of different polarity, including in boiling water. All these features made URJC-1 a very attractive candidate for catalytic applications. Acid catalytic properties of URJC-1 material were demonstrated in Friedel–Crafts acylation of anisole with acetyl chloride, whereas its basic character was evidenced from the base-catalyzed Knoevenagel condensation of benzaldehyde with malononitrile. In both reactions, the URJC-1 material showed a better catalytic performance than other MOF materials, like commercial HKUST-1 (Basolite C-300) and UiO-66-NH_2_, this one having acid and basic properties too, and also better than conventional zeolitic materials for the case of Friedel–Crafts acylation. Moreover, URJC-1 was stable under the reaction conditions of both catalytic tests, even after several consecutive reaction cycles.

## Supplementary Materials

The following are available online at www.mdpi.com/2073-4360/8/2/44/s1. Crystallographic data of URJC-1 material. Figure S1: URJC-1 structure from checkcif file; Figure S2: Structural details of URJC-1’s pore system; Table S1: Crystal data and structure refinement for compound URJC-1; Table S2: TOF parameter of different catalysts after 1 h of anisole acylation reaction; Table S3: TOF parameter of different catalysts after 1 h of Knoevenagel reaction.
