# Supplementary material for: New URJC-1 Material with Remarkable Stability and Acid-Base Catalytic Properties
Source: Polymers (Basel). 2016 Feb 5;8(2):44. doi: 10.3390/polym8020044 (PMC6432527; doi:10.3390/polym8020044)
Supplement: Supplementary file 1 [file polymers-08-00044-s001.pdf]

Supplementary Materials: New URJC-1 Material with Remarkable Stability and Acid-Base Catalytic Properties

Pedro Leo, Fernando Martínez, Guillermo Calleja, David Briones, Lukasz Wojtas and Gisela Orcajo

S1. Crystallographic Data of URJC-1 Material

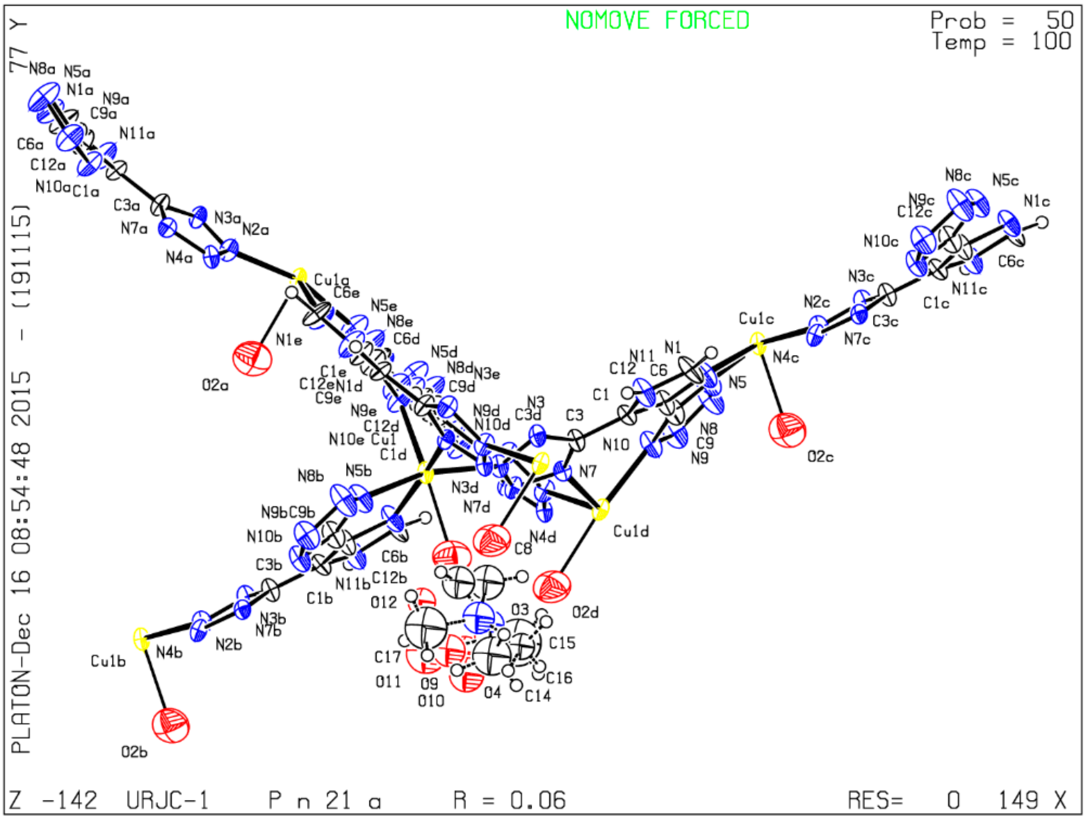

Figure S1. URJC-1 structure from checkcif file.

Table S1. Crystal data and structure refinement for compound URJC-1.

Identification code	URJC-1
Empirical formula	$C_{6.5}H_{5.5}CuN_{10.5}O_{1.25}$
Formula weight	314.25
Temperature/K	100.0
Crystal system	orthorhombic
Space group	$Pn2_1a$
a/Å	8.6577(6)
b/Å	9.0478(9)
c/Å	14.9973(11)
α/°	90
β/°	90
γ/°	90
Volume/Å^3	1,174.79(17)
Z	4
ρ_{calc}g/cm^3	1.777
μ/mm^{-1}	2.811
F(000)	628.0
Crystal size/mm^3	0.02 × 0.02 × 0.02
Radiation	CuK*α* (*λ* = 1.54178)
2θ range for data collection/°	11.802 to 136.714
Index ranges	−10 ≤ h ≤ 9, −10 ≤ k ≤ 10, −17 ≤ l ≤ 17
Reflections collected	7090
Independent reflections	1999 [R_{int} = 0.1146, R_{sigma} = 0.0795]
Data/restraints/parameters	1,999/101/210
Goodness-of-fit on F^2	1.127
Final R indexes [*I*>=2σ (I)]	R_1 = 0.0619, wR_2 = 0.1311
Final R indexes [all data]	R_1 = 0.0801, wR_2 = 0.1399
Largest diff. peak/hole/e Å^{-3}	0.39/−0.46
Flack parameter	0.20(7)

S2. Structural Details of URJC-1 Structure

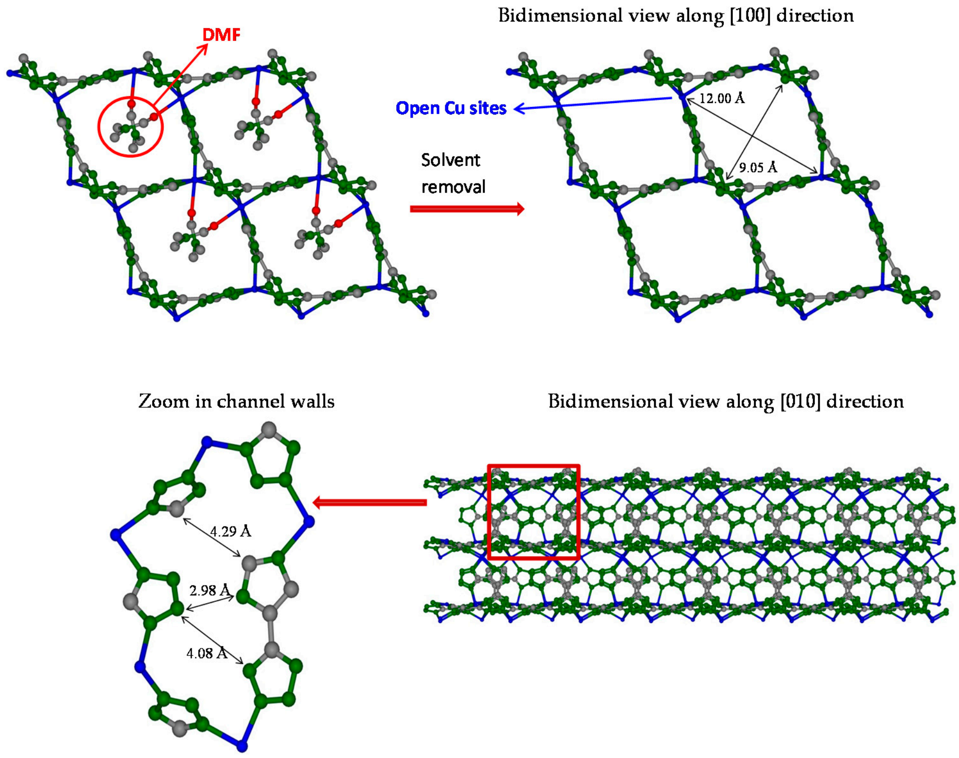

Figure S2. Structural details of URJC-1's pore system.

S3. Reaction Turnover Frequency Parameters (TOF)

Table S2. TOF parameter of different catalysts after 1 h of anisole acylation reaction.

Catalyst	TOF (h^{-1})	Active sites [a] (mmol/mg)	Si/Al	Reference
URJC-1	47	0.0038	-	This work
HKUST-1 [b]	24	0.0050	-	This work
ZSM-5 [b]	76	0.00053	30	This work
BEA ZEOLITE[b]	110	0.00083	19	This work
ZSM-5[c]	26	0.00053	31	[30]

[a] Aluminum atoms in the case of zeolites, and copper atoms in the case of URJC-1 and HKUST-1 MOF materials; [b] HKUST-1, ZSM-5, and BETA were purchased to Sigma–Aldrich Química S.L., Sud Chemie Iberia S.L. and Zeolyst International, respectively; [c]: Synthesized by the authors of reference [30].

Table S3. TOF parameter of different catalysts after 1 h of Knoevenagel reaction.

Catalyst	TOF (h^{-1})	Active sites [a] (mmol/mg)
URJC-1	4	0.0038
HKUST-1	19	0.0099
UiO-66-NH_2	83	0.00061

[a] Basic non-coordinated nitrogen atoms from tetrazole rings for URJC-1; basic oxygen atoms from the carboxylate ligand for HKUST-1; basic amino groups for UiO-66-NH_2.

© 2016 by the authors; licensee MDPI, Basel, Switzerland. This article is an open access article distributed under the terms and conditions of the Creative Commons by Attribution (CC-BY) license (http://creativecommons.org/licenses/by/4.0/).
